# Weak base drug-induced endolysosome iron dyshomeostasis controls the generation of reactive oxygen species, mitochondrial depolarization, and cytotoxicity

**DOI:** 10.1515/nipt-2023-0021

**Published:** 2024-01-11

**Authors:** Peter W. Halcrow, Darius N. K. Quansah, Nirmal Kumar, Rebecca L. Solloway, Kayla M. Teigen, Kasumi A. Lee, Braelyn Liang, Jonathan D. Geiger

**Affiliations:** Department of Biomedical Sciences, School of Medicine and Health Sciences, University of North Dakota, Grand Forks, ND, USA

**Keywords:** weak base drugs, ADMET, endolysosome ferrous iron, cell death, mitochondrial membrane potential, reactive oxygen species

## Abstract

**Objectives:**

Approximately 75 % of marketed drugs have the physicochemical property of being weak bases. Weak-base drugs with relatively high pK_a_ values enter acidic organelles including endosomes and lysosomes (endolysosomes), reside in and de-acidify endolysosomes, and induce cytotoxicity. Divalent cations within endolysosomes, including iron, are released upon endolysosome de-acidification. Endolysosomes are “master regulators of iron homeostasis”, and neurodegeneration is linked to ferrous iron (Fe^2+^)-induced reactive oxygen species (ROS) generation via Fenton chemistry. Because endolysosome de-acidification-induced lysosome-stress responses release endolysosome Fe^2+^, it was crucial to determine the mechanisms by which a functionally and structurally diverse group of weak base drugs including atropine, azithromycin, fluoxetine, metoprolol, and tamoxifen influence endolysosomes and cause cell death.

**Methods:**

Using U87MG astrocytoma and SH-SY5Y neuroblastoma cells, we conducted concentration-response relationships for 5 weak-base drugs to determine EC_50_ values. From these curves, we chose pharmacologically and therapeutically relevant concentrations to determine if weak-base drugs induced lysosome-stress responses by de-acidifying endolysosomes, releasing endolysosome Fe^2+^ in sufficient levels to increase cytosolic and mitochondria Fe^2+^ and ROS levels and cell death.

**Results:**

Atropine (anticholinergic), azithromycin (antibiotic), fluoxetine (antidepressant), metoprolol (beta-adrenergic), and tamoxifen (anti-estrogen) at pharmacologically and therapeutically relevant concentrations (1) de-acidified endolysosomes, (2) decreased Fe^2+^ levels in endolysosomes, (3) increased Fe^2+^ and ROS levels in cytosol and mitochondria, (4) induced mitochondrial membrane potential depolarization, and (5) increased cell death; effects prevented by the endocytosed iron-chelator deferoxamine.

**Conclusions:**

Weak-base pharmaceuticals induce lysosome-stress responses that may affect their safety profiles; a better understanding of weak-base drugs on Fe^2+^ interorganellar signaling may improve pharmacotherapeutics.

## Introduction

Most pharmaceutical drugs have the physicochemical property of being weak bases with relatively high pK_a_ values [1]. Weak base pharmaceuticals have favorable pharmacokinetic properties for absorption, distribution, metabolism, excretion and toxicity (ADMET) [2, 3]. However, weak base drugs can also enter acidic organelles including endosomes and lysosomes (endolysosomes) wherein they cause lysosome stress responses including de-acidification [4,5]. Although pH-driven ion trapping of weak base drugs in endolysosomes is known [6,7], the extent to which and mechanisms by which weak base drugs affect inter-organellar signaling and induce cytotoxicity remain unclear.

Endolysosomes contain high concentrations of readily-releasable ferrous iron (Fe^2+^) and are central to iron trafficking and redox signaling [8, 9]. Drug-induced cytotoxicity and the pathogenesis of diverse disease states are linked to increased iron levels and iron dysregulation [10, 11]; Fe^2+^ via Fenton-like chemistry generates reactive oxygen species (ROS) and induces cytotoxicity [12]. Furthermore, endolysosome Fe^2+^ release induced by endolysosome de-acidification causes iron dysregulation [13], [14], [15]. We reported recently in numerous cell types that de-acidification of endolysosomes decreased Fe^2+^ levels in endolysosomes, increased Fe^2+^ and ROS levels in the cytosol and mitochondria, as well as induced cell death [14], [15], [16]. Because endolysosome sequestration of weak base drugs can result in endolysosome de-acidification [17], we tested the hypothesis that ion trapping of weak base drugs in endolysosomes might be a mechanism common to changes in inter-organellar signaling and increases in cytotoxicity caused by weak base drugs.

The functionally and structurally diverse group of weak base drugs chosen for this study (listed in alphabetical order) were atropine, azithromycin, fluoxetine, metoprolol, and tamoxifen [18], [19], [20], [21], [22]. These drugs were chosen because (1) they are therapeutically beneficial, (2) they have been shown to cause adverse effects, and (3) they readily and effectively cross the blood brain barrier and reach concentrations much higher than those used in our studies [23], [24], [25], [26], [27], [28]. Atropine (pK_a_=10.3), an anti-cholinergic, induces mitochondrial dysfunction and apoptosis [29]. Azithromycin (pK_a_=8.5), a broad-spectrum antibiotic, induces cardiovascular cell death [30]. Fluoxetine (pK_a_=9.8), an anti-depressant, increases cytosolic ROS levels and cell death [31]. Metoprolol (pK_a_=9.7), a beta adrenergic blocker, induces toxicity and acute myocardial reinfarctions [32]. Tamoxifen (pK_a_=8.8), an anti-cancer drug, induces apoptosis [33].

Here, using U87MG astrocytoma and SH-SY5Y neuroblastoma cells, we demonstrated that pharmacologically and therapeutically relevant doses of atropine, azithromycin, fluoxetine, metoprolol, and tamoxifen de-acidified endolysosomes, decreased Fe^2+^ levels in endolysosomes, increased Fe^2+^ and ROS levels in the cytosol and mitochondria, induced mitochondrial membrane potential depolarization, and increased cell death; effects all prevented by the endocytosed iron-chelator deferoxamine. Thus, the weak base drug-induced increases in Fe^2+^ and ROS levels in the cytosol and mitochondria as well as cell death appear downstream of Fe^2+^ released from endolysosomes induced by endolysosome de-acidification. Increased understanding of weak base drug-induced alterations of Fe^2+^ signaling between organelles may lead to improved pharmacotherapeutics and novel insights into adverse effects of commonly used drugs and disease pathogenesis.

## Materials and methods

The experiments performed in this manuscript were not pre-registered. No blinding procedures were performed. No statistical method was employed to pre-determine the sample size of the experiments. No randomization methods were used.

### Cell cultures

Human neuroblastoma cells (SH-SY5Y) and astrocytoma cells (U87MG) were cultured in Dulbecco’s Modified Eagle Medium (Invitrogen, cat. no. 11995, Carlsbad, USA) containing 10 % fetal bovine serum (ThermoFisher, Waltham, USA) and 1 % penicillin/streptomycin (Invitrogen, cat. no. 15140122, Carlsbad, USA). SH-SY5Y cells and U87MG cells were grown in T75 flasks and sub-cultured as needed to 70–80 % confluency. Cells were passaged every 3–4 days using 0.025 % trypsin (Invitrogen, Carlsbad, USA) and maintained in an incubator set at 37 °C and 5 % CO_2_. Cells were not used past their 10th passage. The cells used in this manuscript are not listed as a commonly misidentified cell line by the International Cell Line Authentication Committee and no authentication experiments were conducted.

### Endolysosome pH

Endolysosome pH was determined using LysoSensor Yellow/Blue DND-160 (ThermoFisher, cat. no. L7545, Waltham, USA), a ratiometric dual-excitation dye. SH-SY5Y cells were grown in 35 mm^2^ dishes overnight and then incubated for 5 min at 37 °C with media containing 10 µM DND-160. After incubation, the media was replaced with new media and then images were taken. During imaging, various drug treatments were added gently by pipetting. Light emission at a wavelength of 520 nm in response to excitation for 2 ms at 340/380 nm was measured every 30 s using a filter-based microscope imaging system (Zeiss, Germany). Using a standard curve, we measured the pH as previously described [34] and we calculated changes in proton concentrations within endolysosomes using the formula pH=−log[H^+^].

### Endolysosome iron

Labile ferrous iron (Fe^2+^) in endolysosomes was detected using the fluorescence probe FeRhoNox-1 (Goryo Chemicals, cat no. GC901, Darmstadt, Germany). We have previously demonstrated that the FeRhoNox-1 dye does not significantly colocalize in Golgi, but rather significantly colocalizes in endolysosomes with a high Pearson’s correlation coefficient and also that FeRhoNox-1 is a specific marker for Fe^2+^ [9]. SH-SY5Y cells were added to 35 mm^2^ dishes at a density of 7 × 10^4^ cells/dish and incubated overnight prior to being taken for experimentation. Cells were incubated with 10 µM FeRhoNox-1 at 37 °C for 1 h, stained with LysoTracker™ Green DND-26 (Invitrogen, cat. no. L7526, Carlsbad, USA) and 1 μg/ml Hoechst 33342 for 10 min, washed three times with warm 1X PBS, and then imaged at an excitation of 532 nm and an emission of 570 nm using our confocal scanning microscope (Zeiss, LSM800). Imaging settings remained the same throughout each set of experiments; images were acquired just prior to (0 h) and 30 min after treatments were applied. Imaris software version 9.7 (Oxford Instruments, Concord, USA) was used to reconstruct the images and data were represented as mean fluorescence intensity (MFI). Endolysosome iron levels were also measured in dissociated SH-SY5Y and U87MG cells using flow cytometry. After treatment and staining, cells were washed three times with warm 1X PBS, dissociated from each well in a 24-well plate by gentle trituration, and then placed in Eppendorf tubes for flow cytometry measurements. FeRhoNox-1 fluorescence measurements were made at an excitation of 532 nm and an emission of 570 nm using our flow cytometer (Attune NxT, ThermoFisher, Waltham, USA).

### Cytosolic iron

Cytosolic Fe^2+^ levels were determined with Phen Green™ SK, diacetate (PGSK) (Invitrogen, cat. no. P14313, Carlsbad, USA). Although PGSK reacts with a variety of metals including Fe^2+^ (Invitrogen), our use of the endolysosome iron-chelator deferoxamine (DFO) minimizes this concern because DFO binds with a high affinity to ferric iron and not those other metals. Therefore, although it is likely that other cations are released from endolysosomes, it appears clear that weak base drug-induced release of iron from endolysosomes quenches PGSK fluorescence. In addition, we have been working (and publishing) with deferoxamine (DFO) for years. We have used this drug in multiple cell lines and primary cultured cells including rat, human, and mouse neurons [9, 16, 35]. We have tested multiple concentrations of DFO and used the chosen concentration of DFO that was effective yet didn’t kill the cells. SH-SY5Y cells and U87MG cells cultured in 24-well plates were first stained with 10 µM PGSK for 20 min in complete media. After 20 min, cells were washed two times with PBS and fresh media was added to the wells. Then cells were incubated with weak base drugs for 1 h. Deferoxamine (50 µM, DFO) was added to the cells 1 h before the addition of weak base drugs. After 1 h incubation, cells were then washed three times with warm 1X PBS to remove unloaded dye, were dissociated from their culture plates by gentle trituration, and placed in Eppendorf tubes where the fluorescence was measured at an excitation of 488 nm and an emission of 530 nm using a flow cytometer (Attune NxT, ThermoFisher, Waltham, USA). Unstained cells were used as controls for background fluorescence. Mean fluorescence intensity (MFI) data were collected from a minimum of 10,000 cells per condition. Because PGSK is a quenching dye, the fluorescence data were transformed and illustrated as the reciprocal of mean fluorescence intensity (1/MFI) to demonstrate the increase in cytosolic iron.

### Cytosolic ROS

Levels of cytosolic ROS were measured using approximately 6 × 10^5^ SH-SY5Y cells/well following preincubation with weak base drugs in the absence or presence of DFO (50 µM) for 1 h followed by 1 h incubations with various treatments. Following incubations, cells were washed three-times with PBS and 10 µM CM-H_2_DCFDA (5-(and-6)-chloromethyl-2′,7′-dichlorodihydrofluorescein diacetate) (Invitrogen, cat. no. C6827, Carlsbad, USA), which is an effective dye for hydroxyl radicals that are a product of the Fe^2+^ + H_2_O_2_ reactants in Fenton’s reaction [36] was added into wells containing PBS. Cells were then incubated for 20 min at 37 °C and 5 % CO_2_; cells were then washed three times with PBS to remove extracellular stain. Cells were then dissociated from their culture plates by gentle trituration, and placed in Eppendorf tubes where the fluorescence was measured at an excitation of 488 nm and an emission of 530 nm using a flow cytometer (Attune NxT, ThermoFisher, Waltham, USA). Unstained cells were used as negative controls to establish background fluorescence. Mean fluorescence intensity (MFI) was acquired on a minimum of 10,000 cells per condition using an Attune NxT flow cytometer (ThermoFisher, Waltham, USA).

### Mitochondrial iron

Mitochondrial Fe^2+^ levels were measured using rhodamine B 4-[(2,2′-bipyridin-4-yl) aminocarbonyl] benzyl ester (RDA) (Guidechem, cat. no. 952228-30-7, Milwaukee, USA). SH-SY5Y cells and U87MG cells were added at a density of about 6 × 10^5^ cells/well to 24-well plates and incubated at 37 °C and 5 % CO_2_ overnight prior to preincubation with weak base drugs for 1 h in the absence and presence of DFO (50 µM) for 1 h. Following treatments, cells were washed three times with warm 1X PBS and incubated with 100 nM RDA for 20 min in PBS in a 37 °C and 5 % CO_2_ incubator_._ Cells were washed three times with PBS to remove extracellular dye. Cells were then dissociated from their culture plates by gentle trituration and then placed in Eppendorf tubes where the fluorescence was measured using a flow cytometer (Attune NxT, ThermoFisher, Waltham, USA). A minimum of 10,000 cells were collected and RDA fluorescence was analyzed at an excitation of 562 nm and an emission of 598 nm. Unstained cells were used to establish background fluorescence and stained cells (without any treatment) were used as buffer controls. Mean fluorescence intensity (MFI) was determined by flow cytometry (Attune NxT, ThermoFisher, Waltham, USA). Because RDA is a quenching dye, the fluorescence data were transformed and illustrated as the reciprocal of mean fluorescence intensity (1/MFI) to demonstrate the increase in mitochondrial iron.

### Mitochondrial ROS

SH-SY5Y cells (6 × 10^5^ cells/well) were added into 12-well plates and incubated at 37 °C and 5 % CO_2_ overnight prior to being taken for experimentation. Cells were preincubated with weak base drugs in the absence or presence of DFO (50 µM) for 1 h, washed gently three-times with PBS, and incubated with 2.5 µM of MitoSox (Invitrogen, cat. no. M36008, Carlsbad, USA), which detects superoxide (O_2_^−^·) in mitochondria and is a product of the Fenton-like reaction Fe^2+^ + O_2_ → O_2_^−^· [37]. MitoSox and the cells were incubated for 20 min in a 37 °C and 5 % CO_2_ incubator. Cells were rinsed three times with warm 1X PBS, fresh PBS was added to the wells, and cells were then dissociated from their culture plates by gentle trituration and then placed in Eppendorf tubes where then the fluorescence was measured using a flow cytometer (Attune NxT, ThermoFisher, Waltham, USA). A minimum of 10,000 cells per condition were collected, and MitoSox fluorescence was analyzed in cells dissociated from the culture plates at an excitation of 510 nm and an emission of 580 nm by flow cytometry (Attune NxT, ThermoFisher, Waltham, USA).

### Mitochondrial membrane potential

SH-SY5Y cells were added to 24-well plates at a density of 6 × 10^5^ cells/well and were incubated overnight at 37 °C and 5 % CO_2_. Cells were preincubated with weak base drugs in the absence or presence of DFO (50 µM), washed gently three-times, and treated with 100 nM MitoTracker™ Red CMXROS (Invitrogen, cat. no. M7512, Carlsbad, USA) for 30 min in a 37 °C and 5 % CO_2_ incubator. Cells were washed three times with warm 1X PBS, re-suspended in 1 ml PBS and dissociated from the culture plates by trituration and placed in Eppendorf tubes and the mean fluorescence intensity was determined using a flow cytometer (Attune NxT, ThermoFisher, Waltham, USA) at an excitation of 579 nm and an emission of 599 nm. For confocal microscopy, SH-SY5Y cells were added to 35 mm^2^ dishes and incubated overnight in an incubator kept at 37 °C and 5 % CO_2_. Cells were treated with respective drugs for 1 h, washed gently three-times with PBS, and incubated with 100 nM Mito-Tracker Red CMXROS and 200 nM MitoTracker™ Green FM (Invitrogen, cat. no. M7514, Carlsbad, USA) for 30 min in a 37 °C and 5 % CO_2_ incubator. Cells were washed three times with PBS, stained with 1 μg/ml Hoechst 33342 for 10 min, washed with PBS three times, and imaged using confocal microscopy (Zeiss 8000, Jena, Germany) at an excitation of 579 nm and an emission of 599 nm.

### Cell death

Cell viability was determined using propidium iodide (PI) (BD Biosciences, cat. no. 556463, Franklin Lakes, USA) and flow cytometry. SH-SY5Y cells and U87MG cells were added to 24-well plates at a density of about 6 × 10^5^ cells/well, incubated overnight at 37 °C and 5 % CO_2_, and cells were treated with respective drugs for 24 h. DFO was added to the cells 1 h prior to adding the weak base drugs. After 24 h, cells were washed three times with warm 1X PBS and stained with PI (3 µM) at room temperature for 15 min. Cells were then dissociated from their culture plates by gentle trituration and then placed in Eppendorf tubes where the fluorescence was measured at an excitation of 532 nm and an emission of 570 nm using a flow cytometer (Attune NxT, ThermoFisher, Waltham, USA).

### Reagents

Chemical reagents were obtained from ThermoFisher Scientific except as noted above.

### Data and statistical analyses

All data were reported as means ± standard deviation (SD). Statistical significance between controls and treatments (two groups) were analyzed with a two-tailed Student’s *t*-test, and statistical significance between 3 groups or more (multiple groups) were analyzed with one-way ANOVA plus a Tukey’s multiple comparison post-hoc test. A p-value less than 0.05 was considered statistically significant. GraphPad Prism 9.3.1 software was used to perform all statistical analyses. No pre-determined exclusion criteria were performed. Normal distribution of data was assessed for statistical analysis by using the Shapiro-Wilk test (p<0.05 was considered as not to conform to a normal distribution). The Kruskal–Wallis non-parametric test was used to compare data that had a skewed distribution. The number of cells used for this study was determined based on previous studies [38], [39], [40]. No outliers were detected using ROUT method. Results were based on experiments conducted at least three separate times; each time in triplicate. These technical replicates were used to ensure the reliability of single values.

### Ethical statement

Institutional ethical approval was not required for this study.

## Results

### Concentration-dependent effects of weak base drugs on levels of endolysosome iron

**Figure 1:**
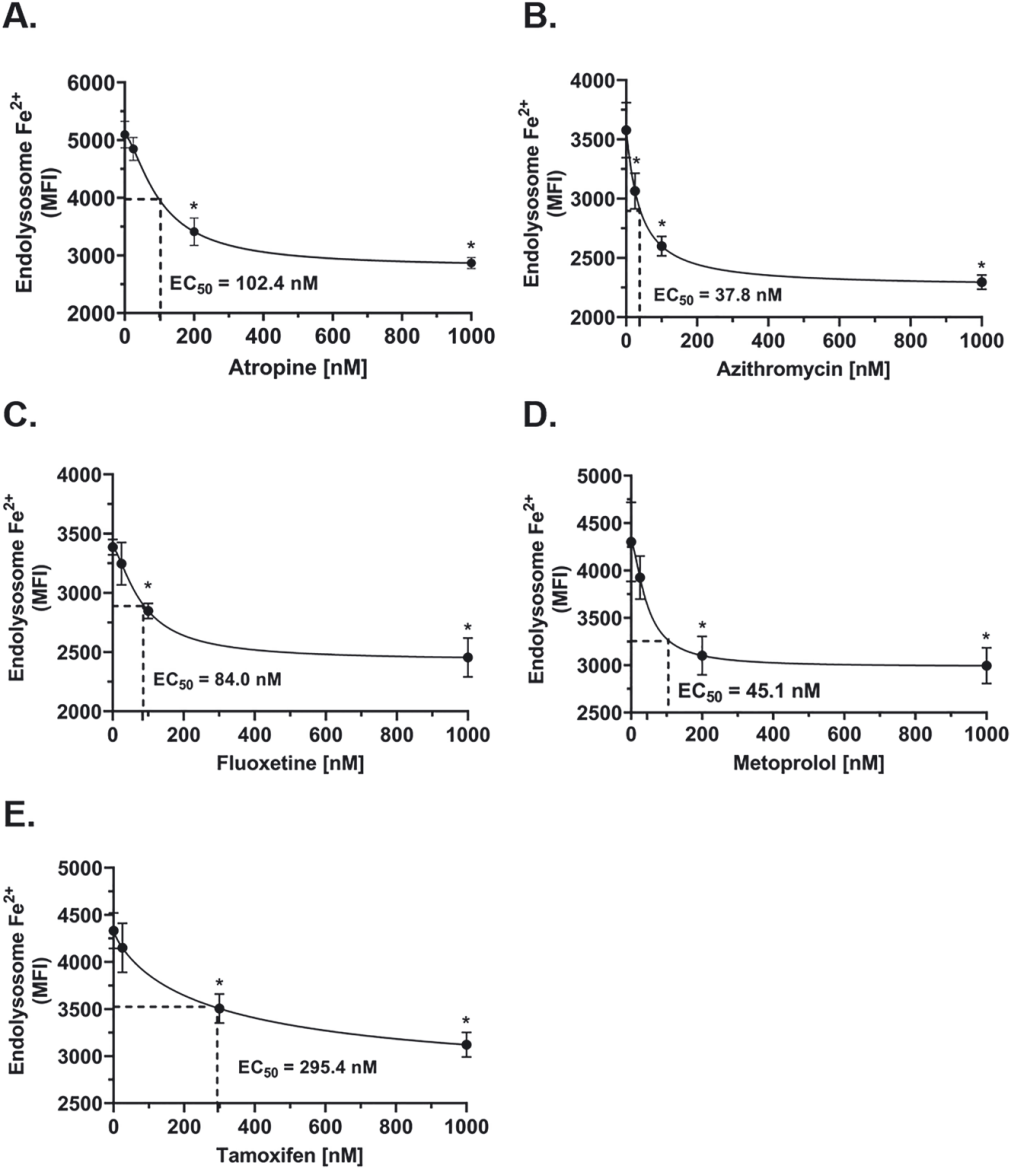
Concentration-dependent effects of weak base drugs on endolysosome iron. (A–E) Using methods we described previously [9, 14], and concentrations similar to those reported to be clinically effective [41], [42], [43], [44], [45], we found that atropine (25, 200, and 1000 nM), azithromycin (25, 100, and 1000 nM), fluoxetine (25, 100, and 1000 nM), metoprolol (25, 200, and 1000 nM), and tamoxifen (25, 300, and 1000 nM) concentration-dependently decreased the fluorescence staining of FeRhoNox-1 for endolysosome Fe^2+^. The calculated EC_50_ values were 102.4 nM for atropine (Figure 1A), 37.8 nM for azithromycin (Figure 1B), 84.0 nM for fluoxetine (Figure 1C), 45.1 nM for metoprolol (Figure 1D), and 295.4 nM for tamoxifen (Figure 1E). For analysis, an ANOVA with Tukey’s post hoc multiple comparisons test was used. The MFI of 10,000 cells was obtained independently from five cell-culture preparations for each drug concentration (n=50,000). For each drug concentration, n=total number of cells. With 50,000 cells used for experimentation per drug concentration, no cells were intentionally excluded. ^*^p<0.05.

**Figure 2:**
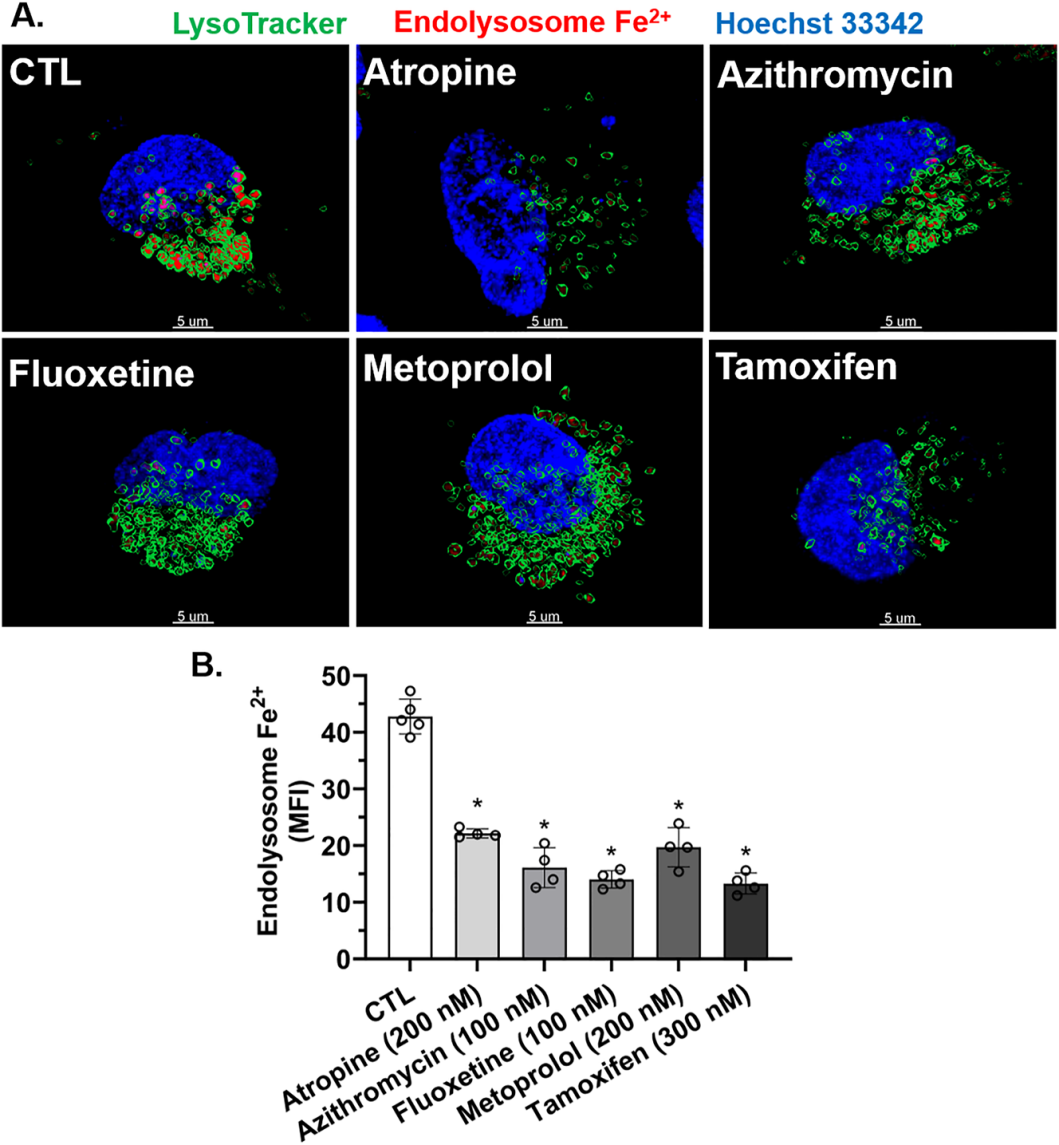
Atropine-, azithromycin-, fluoxetine-, metoprolol-, and tamoxifen-induced decreases in endolysosome Fe^2^ levels. (A) Qualitatively, atropine, azithromycin, fluoxetine, metoprolol, and tamoxifen decreased the fluorescence staining of FeRhoNox-1 for endolysosome Fe^2+^. Images were acquired with a confocal spinning-disc microscope of SH-SY5Y cells that were treated for a 30 min period with water (CTL), atropine (200 nM), azithromycin (100 nM), fluoxetine (100 nM), metoprolol (200 nM), and tamoxifen (300 nM). The images were reconstructed with the software Imaris 3D and illustrate FeRhoNox-1 staining (red) of Fe^2+^ inside of endolysosomes. (B) Quantitatively, endolysosome Fe^2+^ levels as indicated by mean fluorescence intensity (MFI) for the staining of FeRhoNox-1 were significantly (p<0.0001) decreased by atropine (200 nM), azithromycin (100 nM), fluoxetine (100 nM), metoprolol (200 nM), and tamoxifen (300 nM). For analysis, an ANOVA with Tukey’s post hoc multiple comparisons test was used. The MFI of 3000 endolysosomes was measured per cell-culture preparation, which was done independently five separate times for each group (n=15,000). Each one of the five-separate cell-culture preparations per group represents a data point. For each group plotted, n=total number of endolysosomes. With 15,000 endolysosomes used for experimentation per group, cells were randomly chosen in the field of view in the microscope. No cells were intentionally excluded. Scale bar=5 μm, ^*^p<0.05.

We first determined concentration-dependent responses of weak base drugs on levels of endolysosome Fe^2+^. Using methods we described previously [9, 14], and concentrations similar to those reported to be clinically effective [41], [42], [43], [44], [45], we found that atropine, azithromycin, fluoxetine, metoprolol, and tamoxifen concentration-dependently decreased FeRhoNox-1 fluorescence staining for endolysosome Fe^2+^ (Figure 1A–E). The calculated EC_50_ values were determined (Figure 1). From these concentration curves and for all subsequent experiments, we chose the following single drug concentration near the EC_50_ that was relevant both pharmacologically and therapeutically; atropine (200 nM), azithromycin (100 nM), fluoxetine (100 nM), metoprolol (200 nM), and tamoxifen (300 nM). Using semi-quantitative methods we described previously [9, 14], atropine, azithromycin, fluoxetine, metoprolol, and tamoxifen decreased the fluorescence staining of FeRhoNox-1 for Fe^2+^ (Figure 2A). Quantitatively, atropine, azithromycin, fluoxetine, metoprolol, and tamoxifen significantly decreased Fe^2+^ concentration in endolysosomes ([Fe^2+^]_el_) by 53 , 60, 61, 54, and 62 %, respectively (Figure 2B).

### The effects of weak base drugs on endolysosome pH

**Figure 3:**
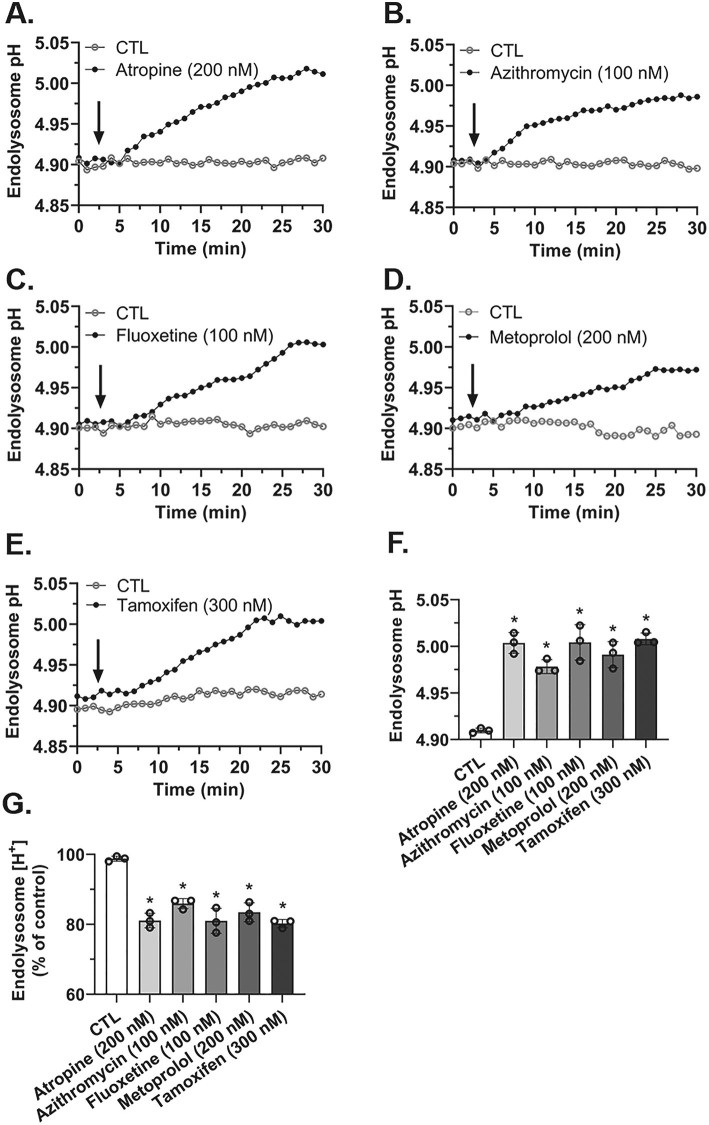
Endolysosome de-acidification by the weak base drugs atropine, azithromycin, fluoxetine, metoprolol, and tamoxifen. (A–E) Representative images of SH-SY5Y cells treated for 30 min with atropine (200 nM), azithromycin (100 nM), fluoxetine (100 nM), metoprolol (200 nM), and tamoxifen (300 nM) showed increased endolysosome pH. (F) Peak levels of endolysosome de-acidification within the 30 min incubation interval were significantly increased by atropine (p<0.0001), azithromycin (p<0.001), fluoxetine (p<0.0001), metoprolol (p<0.0001) and tamoxifen (p<0.0001). (G) Peak levels of endolysosome hydrogen ions within the 30 min incubation interval were significantly (p<0.0001) decreased by atropine, azithromycin, fluoxetine, metoprolol, and tamoxifen. (F and G) Individual data points as well as mean ± SD values were included on each bar. For analysis, an ANOVA with Tukey’s post hoc multiple comparisons test was used. The mean pH measurement of 50 endolysosomes across five cells was obtained independently from five cell-culture preparations for each group (n=250) and represents each data point. For each group plotted, n=total number of endolysosomes. With 20 cells used for experimentation per group, cells were randomly chosen in the field of view in the microscope. No cells were intentionally excluded. *p<0.05.

To confirm previous findings and because endolysosome de-acidification decreases [Fe^2+^]_el_ [9, 14, 18], [19], [20], [21], we next determined the effects of atropine, azithromycin, fluoxetine, metoprolol, and tamoxifen on endolysosome pH. Atropine, azithromycin, fluoxetine, metoprolol, and tamoxifen significantly de-acidified endolysosomes; illustrated in real-time (Figure 3A–E) as well as the peak responses in pH over a duration of 30 min (Figure 3F and G). Endolysosome de-acidification by atropine (Figure 3A), azithromycin (Figure 3B), fluoxetine (Figure 3C), metoprolol (Figure 3D), and tamoxifen (Figure 3E) increased slowly to peak effects over the 30 min incubation period (Figure 3F and G). These pH changes equate to significant decreases in the proton concentrations in endolysosomes of 19 % with atropine (200 nM), 14 % with azithromycin, 19 % with fluoxetine, 17 % with metoprolol, and 20 % with tamoxifen (Figure 3G).

### Weak base drugs increased cytosolic Fe^2+^ and reactive oxygen species levels and these increases were prevented by the endolysosome-specific iron chelator deferoxamine

**Figure 4:**
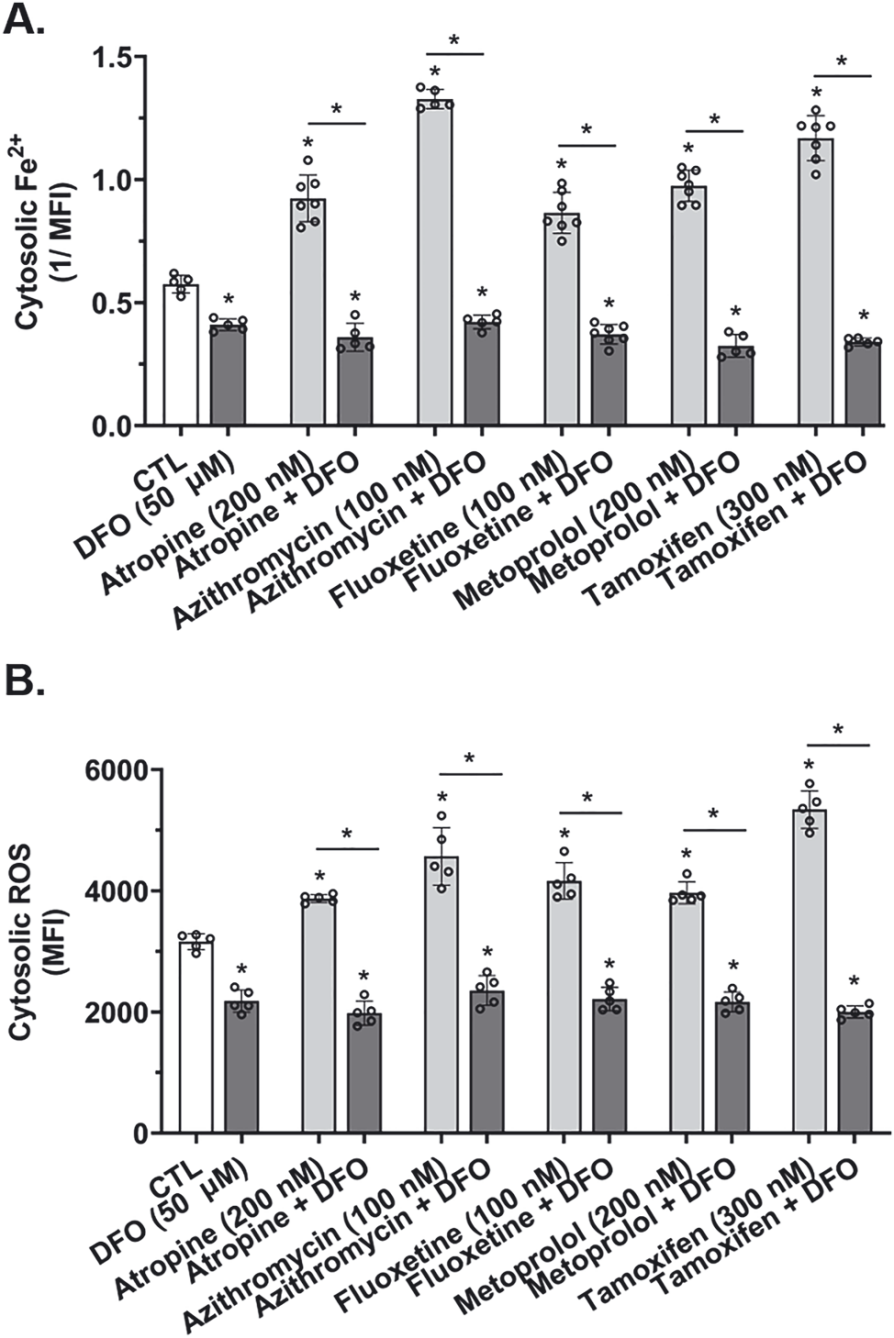
Atropine-, azithromycin-, fluoxetine-, metoprolol-, and tamoxifen-induced increases in cytosolic Fe^2^ and ROS levels were prevented by the endolysosome iron chelator deferoxamine. (A) Cytosolic Fe^2+^ levels were measured using the PhenGreen SK (PGSK) dye that quenches upon binding to Fe^2+^, thus, these data were transformed and illustrated as the reciprocal of mean fluorescence intensity (1/MFI). A 1 h pre-treatment with the endolysosome iron chelator deferoxamine (DFO, 50 µM) significantly (p<0.0001) decreased cytosolic Fe^2+^ levels and significantly (p<0.0001) decreased atropine- (200 nM), azithromycin- (100 nM), fluoxetine- (100 nM), metoprolol- (200 nM), and tamoxifen- (300 nM) induced increases in cytosolic Fe^2+^ levels. (B) Cytosolic ROS levels, including hydroxyl radicals, were measured using 5- (and 6)-chloromethyl-2′,7′-dichlorodihydrofluorescein diacetate acetyl ester (CM-H_2_DCFDA). A 1 h pre-treatment with the endolysosome iron chelator deferoxamine (DFO, 50 µM) significantly (p<0.0001) decreased cytosolic ROS levels and significantly (p<0.0001) decreased atropine- (200 nM), azithromycin- (100 nM), fluoxetine- (100 nM), metoprolol- (200 nM), and tamoxifen- (300 nM) induced increases in cytosolic ROS levels. For analysis, an ANOVA with Tukey’s post hoc multiple comparisons test was used. The MFI of 10,000 cells was measured per cell-culture preparation, which was done independently five-to-seven separate times for each group (n=50,000–70,000). Each one of the five-to-seven separate cell-culture preparations per group represents a data point. For each group plotted, n=total number of cells. With 50,000–70,000 cells used for experimentation per group, no cells were intentionally excluded. ^*^p<0.05.

Endogenous “labile” or “free” iron exists mostly as Fe^2+^ and ferritin complexes with several thousand molecules of ferric (Fe^3+^) iron inside a protein cage [46, 47]. PhenGreen SK is a cytosolically-diffused phenanthroline-based fluorescent probe for cytosolic Fe^2+^ [48], is more sensitive than is calcein for detecting intracellular Fe^2+^, and binds with very high affinity intracellular Fe^2+^ [46, 48, 49]. Thus, others and we use PhenGreen SK with confidence to measure effects of various treatments on levels of cytosolic Fe^2+^. Previously, we reported that endolysosome stores of Fe^2+^ when released were sufficiently large enough to account for insult-induced increases in cytosolic Fe^2+^ levels ([Fe^2+^]_cyt_) and in reactive oxygen species (ROS) levels [14, 15]. Accordingly, we next investigated the extent to which atropine, azithromycin, fluoxetine, metoprolol, and tamoxifen increased levels of [Fe^2+^]_cyt_ (Figure 4A) and cytosolic ROS (Figure 4B). Atropine, azithromycin, fluoxetine, metoprolol, and tamoxifen significantly increased [Fe^2+^]_cyt_ (Figure 4A) and levels of cytosolic ROS (Figure 4B). Consistent with our previously published findings [14, 15], DFO (50 µM) alone significantly decreased [Fe^2+^]_cyt_ (Figure 4A); findings that suggest to us that there is an endogenous leak of Fe^2+^ from endolysosomes. DFO also significantly decreased cytosolic ROS levels (Figure 4B). DFO pre-treatment significantly blocked atropine-, azithromycin-, fluoxetine-, metoprolol-, and tamoxifen-induced increases in [Fe^2+^]_cyt_ (Figure 4A) as well as increases in ROS levels in the cytosol (Figure 4B). Similar effects were observed in U87MG astrocytoma cells in which DFO (50 µM) pre-treatment significantly blocked atropine-, azithromycin-, fluoxetine-, metoprolol-, and tamoxifen-induced increases in [Fe^2+^]_cyt_ (Supplemental Figure 1).

### Weak base drug-induced increases in mitochondrial Fe^2+^ and reactive oxygen species levels were prevented by the endolysosome-specific iron chelator deferoxamine

**Figure 5:**
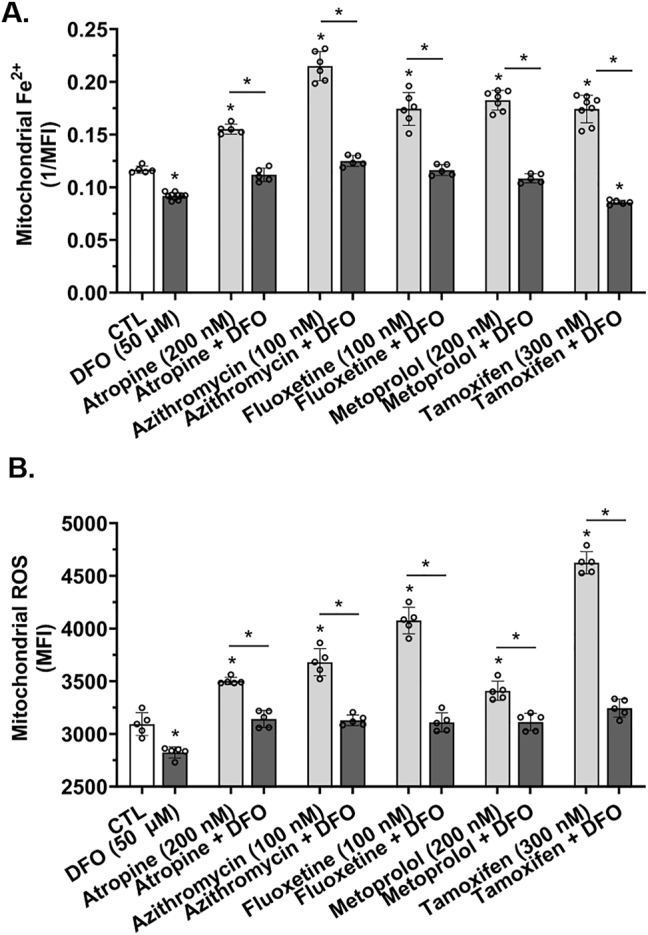
Atropine-, azithromycin-, fluoxetine-, metoprolol-, and tamoxifen-induced increases in mitochondrial Fe^2^ and ROS levels were prevented by the endolysosome iron chelator deferoxamine. (A) Mitochondrial Fe^2^ levels ([Fe^2+^]_mito_) were measured using the rhodamine B-[(2,2′-bipyridine-4-yl)-aminocarbonyl]benzyl ester (RDA) dye that quenches upon binding to Fe^2+^, thus, these data were transformed and illustrated as the reciprocal of mean fluorescence intensity (1/MFI). A 1 h pre-treatment with the endolysosome iron chelator deferoxamine (DFO, 50 µM) significantly (p<0.001) decreased [Fe^2+^]_mito_ and significantly (p<0.0001) decreased atropine- (200 nM), azithromycin- (100 nM), fluoxetine- (100 nM), metoprolol- (200 nM), and tamoxifen- (300 nM) induced increases in [Fe^2+^]_mito_. (B) Mitochondrial ROS levels were determined using MitoSox, and data were expressed as MFI. A 1 h pre-treatment with the endolysosome iron chelator deferoxamine (DFO, 50 µM) significantly decreased mitochondrial ROS levels and significantly (p<0.0001) decreased atropine- (200 nM), azithromycin- (100 nM), fluoxetine- (100 nM), metoprolol- (200 nM), and tamoxifen- (300 nM) induced increases mitochondrial ROS levels. For analysis, an ANOVA with Tukey’s post hoc multiple comparisons test was used. The MFI of 10,000 cells was measured per cell-culture preparation, which was done independently five-to-seven separate times for each group (n=50,000–70,000). Each one of the five-to-seven separate cell-culture preparations per group represents a data point. For each group plotted, n=total number of cells. With 50,000–70,000 cells used for experimentation per group, no cells were intentionally excluded. ^*^p<0.05.

Endolysosome Fe^2+^ stores, when released, are sufficiently large to increase concentrations of mitochondrial Fe^2+^ ([Fe^2+^]_mito_) and levels of ROS [9, 14, 15]. Accordingly, we next investigated the extent to which atropine, azithromycin, fluoxetine, metoprolol, and tamoxifen increased [Fe^2+^]_mito_ and levels of mitochondrial ROS. Atropine, azithromycin, fluoxetine, metoprolol, and tamoxifen significantly increased [Fe^2+^]_mito_ (Figure 5A) and significantly increased mitochondrial ROS levels (Figure 5B). [Fe^2+^]_mito_ and mitochondrial ROS levels were significantly decreased by DFO (50 µM) alone (Figure 5A and B). DFO pre-treatment significantly blocked atropine-, azithromycin-, fluoxetine-, metoprolol-, and tamoxifen-induced increases in [Fe^2+^]_mito_ (Figure 5A) as well as increases in mitochondrial ROS levels (Figure 5B). Similar effects were observed in U87MG astrocytoma cells; DFO pre-treatment significantly blocked atropine-, azithromycin-, fluoxetine-, metoprolol-, and tamoxifen-induced increases in [Fe^2+^]_mito_ (Supplemental Figure 2).

### Weak base drug-induced depolarization of mitochondrial membrane potential was prevented by the endolysosome-specific iron chelator deferoxamine

**Figure 6:**
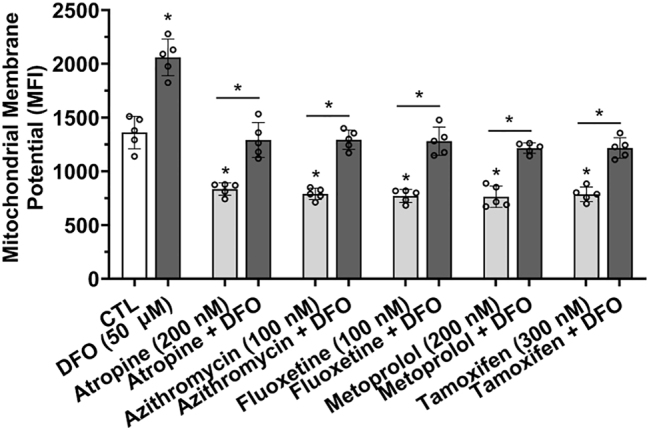
Weak base drug-induced depolarization of mitochondrial membrane potential was prevented by the endolysosome iron chelator deferoxamine. The dye CMXROS was used to measure mitochondrial membrane potential (∆*ψ*_m_) where the intensity of the dye decreases when the ∆*ψ*_m_ is depolarized. A 1 h pre-treatment with the endolysosome iron chelator deferoxamine (DFO, 50 µM) significantly (p<0.0001) polarized the mitochondrial membrane potential and significantly (p<0.0001) blocked atropine- (200 nM), azithromycin- (100 nM), fluoxetine- (100 nM), metoprolol- (200 nM), and tamoxifen- (300 nM) induced mitochondrial membrane potential depolarization. For analysis, an ANOVA with Tukey’s post hoc multiple comparisons test was used. The MFI of 10,000 cells were obtained independently from five cell-culture preparations for each group (n=50,000) and represents each data point. For each group plotted, n=total number of cells. With 50,000 cells used for experimentation per group, no cells were intentionally excluded. ^*^p<0.05.

Endolysosome Fe^2+^ stores, when released, are sufficiently large to depolarize mitochondrial membrane potentials [15]. Accordingly, we next determined the effects of weak base drugs on mitochondrial membrane potential (∆*ψ*_m_). A 1 h treatment of SH-SY5Y cells with atropine, azithromycin, fluoxetine, metoprolol, and tamoxifen significantly decreased CMXROS fluorescence staining; findings indicative of ∆*ψ*_m_ depolarization (Figure 6). DFO pre-treatment for 1 h significantly blocked atropine-, azithromycin-, fluoxetine-, metoprolol-, and tamoxifen-induced ∆*ψ*_m_ depolarization, and the ∆*ψ*_m_ was significantly polarized (increased) by DFO alone (Figure 6).

### Weak base drug-induced cell death was prevented by the endolysosome-specific iron chelator deferoxamine

**Figure 7:**
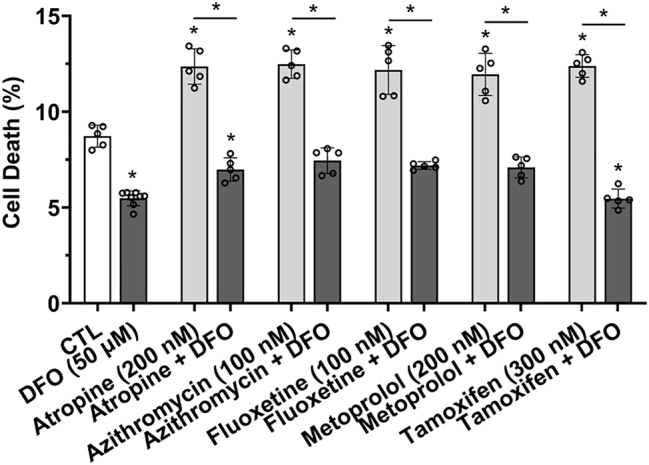
Weak base drug-induced increases in cell death was prevented by the endolysosome iron chelator deferoxamine. Cell death of SH-SY5Y cells was measured using propidium iodide 24 h post-drug treatments. A 1 h pre-treatment with the endolysosome iron chelator deferoxamine (DFO, 50 µM) significantly (p<0.0001) decreased the percentage of cell death and significantly (p<0.0001) blocked atropine- (200 nM), azithromycin- (100 nM), fluoxetine- (100 nM), metoprolol- (200 nM), and tamoxifen- (300 nM) induced increases in the percentage of cell death. For analysis, an ANOVA with Tukey’s post hoc multiple comparisons test was used. The MFI of 10,000 cells were obtained independently from five cell-culture preparations for each group (n=50,000) and represents each data point. For each group plotted, n=total number of cells. With 50,000 cells used for experimentation per group, no cells were intentionally excluded. ^*^p<0.05.

Endolysosome Fe^2+^ stores, when released, are sufficiently large to cause cell death [15, 16]. Accordingly, we next tested the effects of weak base drugs on cell viability using propidium iodide 24 h post-drug treatments. Atropine, azithromycin, fluoxetine, metoprolol, and tamoxifen significantly increased cell death (Figure 7). DFO alone significantly decreased cell death, and DFO pre-treatment for 1 h significantly blocked atropine-, azithromycin-, fluoxetine-, metoprolol-, and tamoxifen-induced cell death (Figure 7). Similar effects were observed in U87MG astrocytoma cells in which DFO pre-treatment for 1 h significantly blocked atropine-, azithromycin-, fluoxetine-, metoprolol-, and tamoxifen-induced cell death (Supplemental Figure 3).

## Discussion and conclusions

**Figure 8:**
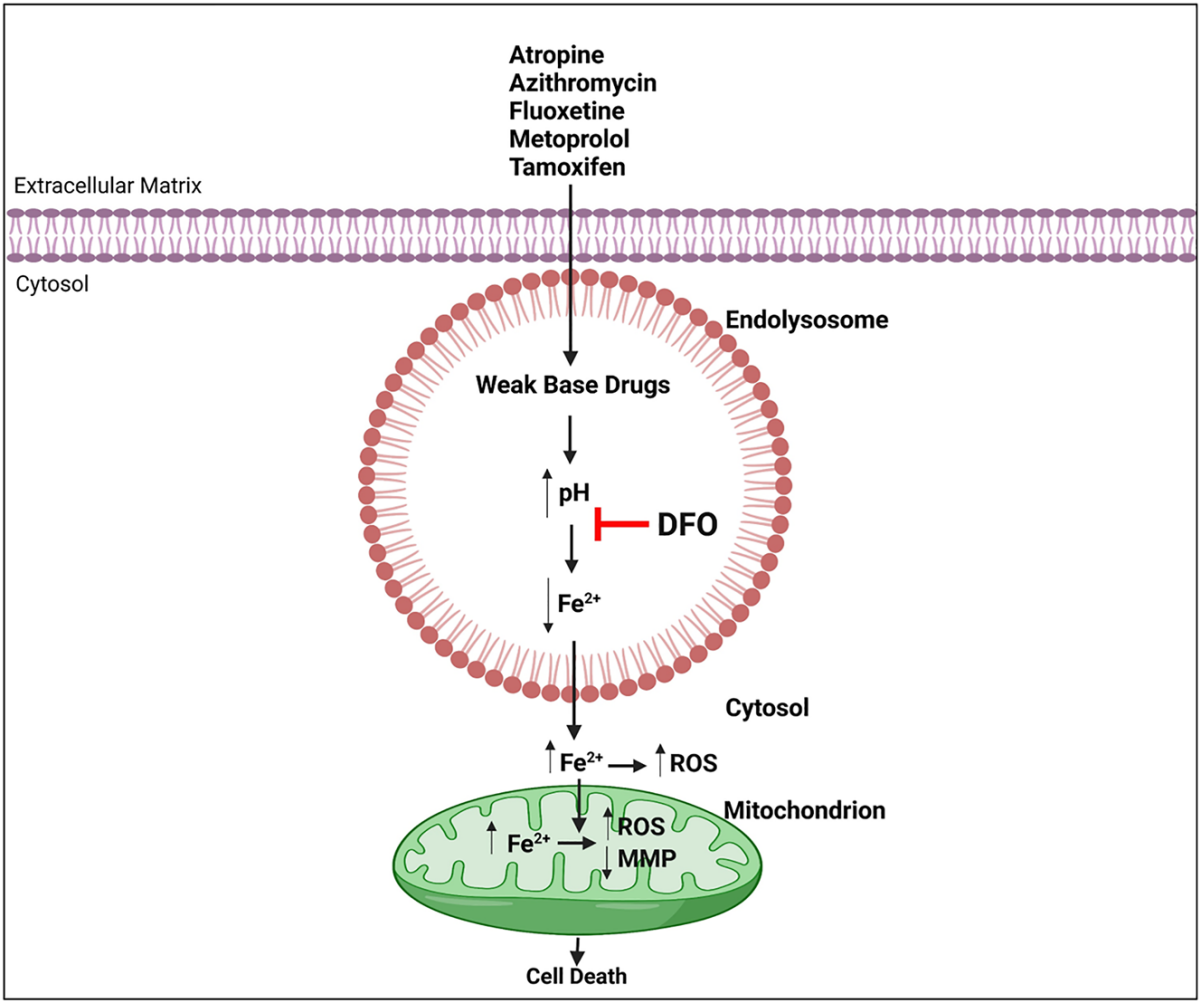
Atropine-, azithromycin-, fluoxetine-, metoprolol-, and tamoxifen-induced endolysosome de-acidification caused an efflux of ferrous iron from endolysosomes. The iron released from endolysosomes was sufficient to increase cytosolic and mitochondria iron and ROS levels, decreased mitochondrial membrane potential (MMP), and increased cell death. Deferoxamine (DFO) blocked the effects of the weak base drugs tested.

It has been estimated that up to 75 % of modern pharmacotherapeutics are weak base drugs that exhibit relatively high pK_a_ values [1, 50]. Although weak base drugs have favorable pharmacokinetic properties and are widely used clinically, they enter endolysosomes where they reside because of ion trapping for extended periods of time, they affect endolysosome structure and function, and they can induce cytotoxicity [1, 6, 7]. Endolysosome sequestration of weak base drugs results in endolysosome de-acidification, and endolysosomes contain high concentrations of readily-releasable Fe^2+^ whereupon inhibiting endolysosome acidity (de-acidification) induces iron dysregulation and endolysosome iron release; endolysosome de-acidification and iron release from endolysosomes have been linked directly to neurotoxicity [9, 14, 51]. Here, using pharmacologically and therapeutically relevant doses of five very different weak base drugs [41], [42], [43], [44], [45], we found that these drugs de-acidified endolysosomes, decreased Fe^2+^ levels in endolysosomes, increased Fe^2+^ and ROS levels in the cytosol and mitochondria, induced mitochondrial membrane potential depolarization, and increased cell death; effects all prevented by the endocytosed iron-chelator deferoxamine (DFO) (Figure 8).

Iron homeostasis is linked to endolysosome acidity, whereupon iron dysregulation is induced by endolysosome de-acidification [13], [14], [15], [16]. Not surprisingly, disruption of endolysosome pH continues to be implicated in neurotoxicity and in a broad range of conditions including cancer, aging, and neurodegenerative diseases [10, 11, 52]. However, despite decades of study, the underlying mechanisms are poorly understood, especially in regards to endolysosomes [11]. Thus, our studies were conducted for time intervals ranging from 30 to 60 min because this is when peak endolysosome responses were observed. These intervals were chosen because we were determining early mechanisms that lead to cell death and not mechanisms affected by cell death.

This functionally and structurally diverse group of weak base drugs was chosen because they are known to de-acidify endolysosomes and induce cytotoxicity [18], [19], [20], [21], [22]. Atropine (pK_a_=10.3) is a clinically relevant anti-cholinergic drug that induces mitochondrial swelling as well as cell death [29]. Azithromycin (pK_a_=8.5) is one of the most commonly prescribed antibiotics and has a known risk for increased cardiovascular cell death [30]. Fluoxetine (pK_a_=9.8) is a type of anti-depressant known as a selective serotonin reuptake inhibitor (SSRI) that increases levels of ROS and induces cell death [31]. Metoprolol (pK_a_=9.7) is a beta-adrenergic blocker that can induce myocardial ischaemia [32]. Tamoxifen (pK_a_=8.8) is an anti-estrogen drug that induces autophagy and cell death [33]. Because these five weak base pharmaceuticals are commonly taken and can provide a therapeutic benefit as well as induce adverse effects, it was important for us to demonstrate the extent to which and mechanisms by which a wide spectrum of weak base drugs affect the pools of endolysosome Fe^2+^.

Previously, we reported that endolysosome de-acidification by the weak base drug chloroquine, bafilomycin which inhibits v-ATPase, the HIV-1 gp120 coat protein, and mu opioid receptor agonists induced endolysosome Fe^2+^ release, increased cytosolic and mitochondrial Fe^2+^ and ROS levels, and increased cell death [14], [15], [16, 53]. Because endolysosomes are important regulators of iron-related neurotoxicity and neurodegeneration, weak base drugs can be neurotoxic because they accumulate in and de-acidify endolysosomes [19, 21, 22]. It was important to determine mechanisms by which a diverse group of weak base drugs including atropine, azithromycin, fluoxetine, metoprolol and tamoxifen induce cytotoxicity and cell death. As demonstrated, these weak base drug-induced increases in Fe^2+^ and ROS levels in the cytosol and mitochondria as well as cell death appear to be downstream events of weak base drug-induced de-acidification of endolysosomes and endolysosome Fe^2+^ release.

The acidic lumen of endolysosomes generates a pH gradient with the cytosol [54]. At physiological pH, the neutral fraction of weak base drugs facilitates drug diffusion through membranes into endolysosome’s acidic lumen whereupon ionization occurs and drug egress into the cytosol is restricted; a process known as ion trapping or lysosomotropism [6]. Notably, 75 percent of drugs are estimated to have weak base pK_a_ values, and the *World Drug Index* (WDI) determined that 63 % of drugs were ionizable between pH 2 and 12 [1, 55]. Furthermore, many cardiovascular and central nervous system drugs are lipophilic amines with amine groups that are ionizable (pK_a_>6); hence, they are lysosomotropic and can accumulate in endolysosomes at concentrations several hundred times higher than the cytosol [56]. Although lipophilic drugs can induce neurotoxicity [57], the entering of weak base drugs via pH partitioning into endolysosome’s acidic lumen initiates cytotoxic responses.

Central to neurotoxicity and neurodegeneration is mitochondrial dysfunction, and many weak base drugs induce mitochondrial dysfunction [58, 59]. In particular, mitochondrial membrane potential (∆*ψ*_m_) depolarization appears to be an early mitochondrial event leading to cell death [60]. Correspondingly, many weak base drugs are known to depolarize the mitochondrial membrane potential [61, 62] and our findings clearly showed that the weak base drug-induced efflux of Fe^2+^ from endolysosomes caused mitochondrial depolarization as well as cell death.

These five weak base drugs readily and effectively cross the blood brain barrier and reach concentrations much higher than those used in our studies. Fluoxetine treatment over 6–8 months in 22 human subjects reached peak brain levels of 10.7 μg/mL; a concentration of 30.9 µM [23]. Another study of 13 human subjects demonstrated that 5 weeks of fluoxetine treatment (20 mg/day) resulted in mean brain tissue concentrations of 25.5 µM and 120 mg/day resulted in mean brain tissue concentrations of 41.4 µM [24]. Tamoxifen treatment (30 mg/day) in human subjects for 7 days resulted in mean concentrations of 1.8 µM in normal brain tissue and 2.7 µM in brain cancerous tissue [25]. Furthermore, a single oral dose of azithromycin (500 mg) in 20 human subjects 48 h prior to surgery resulted in mean concentrations of 5 µM in brain tissue [26]. Human subjects who received metoprolol (200 mg/day) for 22 days had mean brain tissue concentrations of 3.5 µM and human subjects who received metoprolol (200 mg/day) for only 3 days had mean brain tissue concentrations of 2.9 µM [27]. In a mouse model, a single dose (10 mg/kg) of atropine resulted in mean brain tissue concentrations of 144 nM [28]. Thus, the concentrations of these five weak base drugs used and reported in our study are relevant to levels reached with their therapeutic use.

In addition to the U87MG astrocytoma and SH-SY5Y neuroblastoma cell lines used in this study, we have studied these same mechanisms in primary cells including rat cortical neurons [9], human Schwann cells [63], human cortical neurons [64], and mouse hepatocytes (unpublished). In all cases, weak-base drugs de-acidified endolysosomes. These drugs circulate throughout the body and enter the brain at concentrations like those used in this study. Thus, the findings are of pharmacotherapeutic relevance and potential clinical significance. Although we have not studied cardiac myocytes, endothelial cells, or breast cancer cells it is extremely likely that these same effects on pH, iron and ROS would be present given how many different cell types we have studied. Furthermore, we have routinely used positive controls including the weak-base chloroquine and the v-ATPase inhibitor bafilomycin A1 [16, 35] to show comparable levels of toxicity with multiple different insults. The observed levels of toxicity were similar to those reported here for the five weak base drugs. Lastly, we confirmed our findings in both SH-SY5Y neuroblastoma cells and U87MG astrocytoma cells to broaden the impact of our studies. Key experiments were conducted using the U87MG cells as mentioned in the Results section (page 16–18) and the data were included in the Supplemental Material section.

Overall, our study demonstrates that a functionally and structurally diverse group of weak base drugs induce Fe^2+^ release from endolysosomes that is sufficient to significantly increase Fe^2+^ levels in the cytosol and mitochondria as well as induce oxidative stress and cell death. The mechanisms underlying this cytotoxicity described here may lead to a better understanding of weak base pharmacotherapeutics and their potential adverse effects.

## Supplementary Material

Supplementary Material Details
