# Supplementary material for: Weak base drug-induced endolysosome iron dyshomeostasis controls the generation of reactive oxygen species, mitochondrial depolarization, and cytotoxicity
Source: NeuroImmune Pharm Ther. 2024 Jan 11;3(1):33–46. doi: 10.1515/nipt-2023-0021 (PMC10961484; doi:10.1515/nipt-2023-0021)
Supplement: Supplementary file 1 — Supplementary Material Details [file j_nipt-2023-0021_suppl_001.pdf]

Supplemental Material

Weak base drug-induced endolysosome iron dyshomeostasis controls the generation of reactive oxygen species, mitochondrial depolarization, and cytotoxicity

Peter W. Halcrow, Darius N.K. Quansah, Nirmal Kumar, Rebecca L. Solloway, Kayla M. Teigen, Kasumi A. Lee, Braelyn Liang, and Jonathan D. Geiger*

***Address Correspondence to:**

Jonathan D. Geiger, Ph.D.

Chester Fritz Distinguished Professor

Department of Biomedical Sciences

University of North Dakota School of Medicine and Health Sciences

504 Hamline Street, Room 110

Grand Forks, North Dakota 58203

(701) 777-2183 (P); jonathan.geiger@und.edu

Supplemental Figure 1

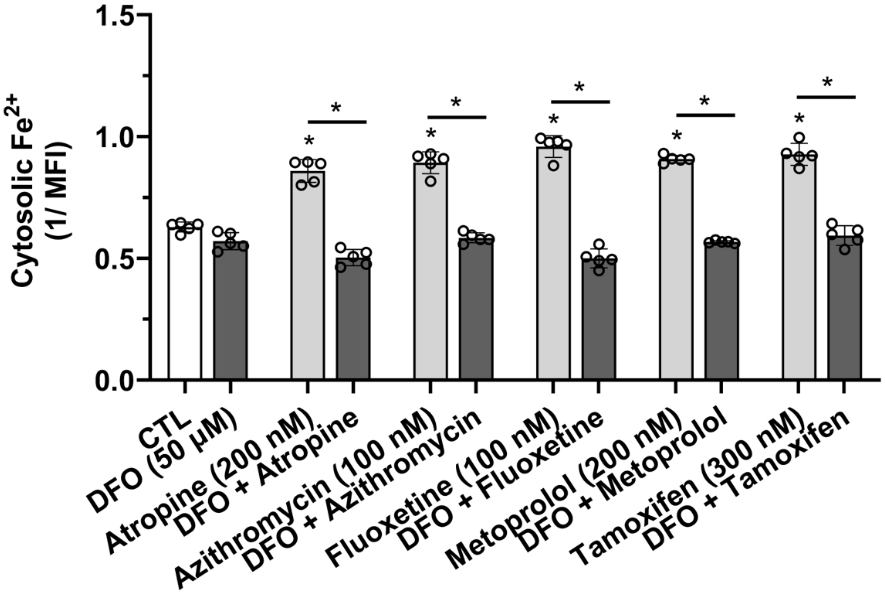

Supplemental Figure 1: Atropine-, azithromycin-, fluoxetine-, metoprolol-, and tamoxifen-induced increases in levels of cytosolic Fe^{2} were blocked by the endolysosome iron chelator deferoxamine. Levels of cytosolic Fe^{2+} in U87MG cells were measured with the quenching dye PhenGreen SK (PGSK), and data were transformed and illustrated as the reciprocal of mean fluorescence intensity (1/MFI). Pre-treatment of cells for 1 h with the endolysosome-specific iron chelator deferoxamine (DFO, 50 µM) significantly ($p<0.0001$) decreased atropine (200 nM), azithromycin (100 nM), fluoxetine (100 nM), metoprolol (200 nM), and tamoxifen (300 nM)-induced increases in levels of cytosolic Fe^{2+}. An ANOVA (Tukey's post hoc test) was used for analysis. Each data point represents the mean fluorescence from 10,000 cells performed independently from five cell-culture preparations for each group (n = 50,000). n = total number of cells for each group plotted. 50,000 cells were used for experimentation per group, and no cells were intentionally excluded. * $p < 0.05$

Supplemental Figure 2

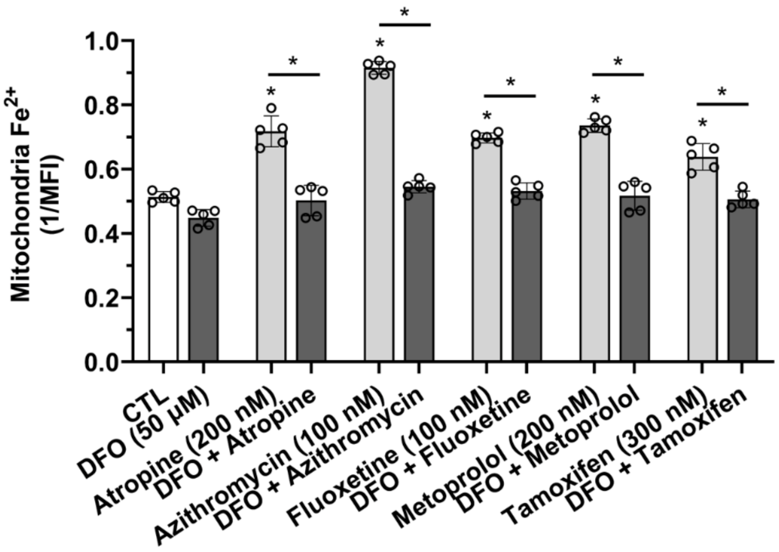

Supplemental Figure 2: Atropine-, azithromycin-, fluoxetine-, metoprolol-, and tamoxifen-induced increases in levels of mitochondrial Fe^2 were blocked by the endolysosome iron chelator deferoxamine. Mitochondrial iron levels ($[Fe^{2+}]_{mito}$) in U87MG cells were determined by using the quenching dye rhodamine B-[(2,2'-bipyridine-4-yl)-aminocarbonyl]benzyl ester (RDA) and fluorescence data were transformed and illustrated as the reciprocal of mean fluorescence intensity (1/MFI). Pre-treatment of cells for 1 h with deferoxamine (DFO, 50 µM) significantly ($p<0.0001$) decreased atropine (200 nM), azithromycin (100 nM), fluoxetine (100 nM), metoprolol (200 nM), and tamoxifen (300 nM)-induced increases in levels of mitochondrial Fe^{2+}. An ANOVA (Tukey's post hoc test) was used for analysis. Each data point represents the mean fluorescence from 10,000 cells performed independently from five cell-culture preparations for each group (n = 50,000). n = total number of cells for each group plotted. 50,000 cells were used for experimentation per group, and no cells were intentionally excluded. * $p < 0.05$

Supplemental Figure 3

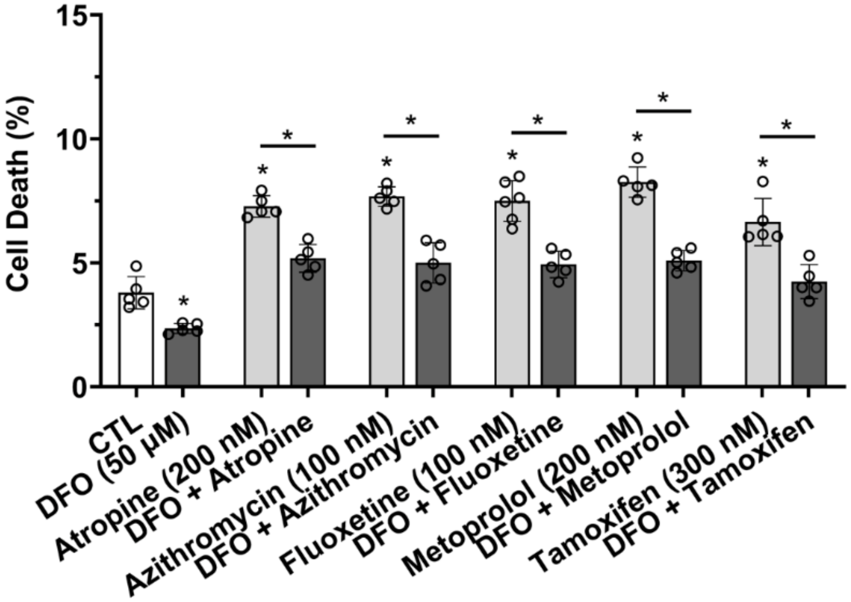

Supplemental Figure 3: Weak base drug-induced increases in cell death were blocked by the endolysosome iron chelator deferoxamine. Propidium iodide was used to measure cell death of U87MG cells after 24 h drug treatments. Pre-treatment of cells for 1 h with the endolysosome-specific iron chelator deferoxamine (DFO, 50 µM) alone significantly ($p<0.05$) decreased the percentage of cell death and significantly ($p<0.0001$) blocked atropine (200 nM), azithromycin (100 nM), fluoxetine (100 nM), metoprolol (200 nM), and tamoxifen (300 nM)-induced increases in the percentage of cell death. An ANOVA (Tukey's post hoc test) was used for analysis. Each data point represents the mean fluorescence from 10,000 cells performed independently from five cell-culture preparations for each group (n = 50,000). n = total number of cells for each group plotted. 50,000 cells were used for experimentation per group, and no cells were intentionally excluded. * $p < 0.05$
